# Serotoninergic Modulation of Basal Cardiovascular Responses and Responses Induced by Isotonic Extracellular Volume Expansion in Rats

**DOI:** 10.5935/abc.20160205

**Published:** 2017-02

**Authors:** Isadora Ferraz Semionatto, Adrieli Oliveira Raminelli, Angelica Cristina Alves, Caroline Santos Capitelli, Rosangela Soares Chriguer

**Affiliations:** 1 Universidade Federal do Triângulo Mineiro (UFTM), Uberaba, MG - Brazil; 2 Universidade Federal de São Paulo (UNIFESP), São Paulo, SP - Brazil

**Keywords:** Serotonin, Serotonin Agents, Rats, Hypotalamic Paraventricular Nucleus, Arterial Pressure, Extracellular Fluid

## Abstract

**Background:**

Isotonic blood volume expansion (BVE) induced alterations of sympathetic and parasympathetic activity in the heart and blood vessels, which can be modulated by serotonergic pathways.

**Objective:**

To evaluate the effect of saline or serotonergic agonist (DOI) administration in the hypothalamic paraventricular nucleus (PVN) on cardiovascular responses after BVE.

**Methods:**

We recorded pulsatile blood pressure through the femoral artery to obtain the mean arterial pressure (MAP), systolic (SBP) and diastolic blood pressure (DBP), heart rate (HR) and the sympathetic-vagal ratio (LF/HF) of Wistar rats before and after they received bilateral microinjections of saline or DOI into the PVN, followed by BVE.

**Results:**

No significant differences were observed in the values of the studied variables in the different treatments from the control group. However, when animals are treated with DOI followed by BVE there is a significant increase in relation to the BE control group in all the studied variables: MBP (114.42±7.85 vs 101.34±9.17); SBP (147.23±14.31 vs 129.39±10.70); DBP (98.01 ±4.91 vs 87.31±8.61); HR (421.02±43.32 vs 356.35±41.99); and LF/HF ratio (2.32±0.80 vs 0.27±0.32).

**Discussion:**

The present study showed that the induction of isotonic BVE did not promote alterations in MAP, HR and LF/HF ratio. On the other hand, the injection of DOI into PVN of the hypothalamus followed by isotonic BVE resulted in a significant increase of all variables.

**Conclusion:**

These results suggest that serotonin induced a neuromodulation in the PVN level, which promotes an inhibition of the baroreflex response to BVE. Therefore, the present study suggests the involvement of the serotonergic system in the modulation of vagal reflex response at PVN in the normotensive rats.

## Introduction

Isotonic blood volume expansion (BVE) induces the activation of several areas of the brain that are important in cardiovascular and neuroendocrine adjustments.^1,2^ BVE activates baroreflex, which promotes hypotension and bradycardia through the excitation of two neural pathways. Hypotension involves excitatory projections of the nucleus of the solitary tract (NST) to the caudal ventrolateral area of the medulla oblongata, and when activated, promotes inhibition of the rostral ventrolateral area of the medulla oblongata, sympathetic-inhibitory pathway, which results in the decrease of sympathetic tone to the heart, reduction of total peripheral resistance, and increase of venous capacitance. Bradycardia involves an excitatory projection of the NST to parasympathetic preganglionic neurons in the dorsal motor nucleus of the vagus and nucleus ambiguous, leading to an increase of vagal efferent in the heart.^3,4^

BVE also promotes an increase in the plasma concentrations of oxytocin (Oxt), mainly synthesized in the paraventricular nucleus (PVN) and in the supraoptic nucleus of the hypothalamus (SON).^4^ Evidence that Oxt acts as an important neuromodulator of the autonomic control of the circulation stemmed from studies in which projections of the PVN to the NST were observed and reinforced by the observation that manipulations in PVN's oxytocinergic system resulted in deep alterations in cardiovascular responses to stress and peptidergic stimuli.^5-8^

The participation of serotonergic mechanisms (5-HT) in PVN responses has also been studied, especially 5-HT action on 5-HT_1A_ and 5-HT_2A_ receptors. Authors have demonstrated the presence of 5-HT_1A_ and 5-HT_2A_ receptors and their respective mRNA in PVN. Moreover, researchers have verified the co-expression of 5-HT_1A_ and 5-HT_2A_ receptors in PVN regions, and a sub-population of these neurons presented double marking, 5-HT_2A_/Oxt, showing evidence of 5-HT participation in the activation of these receptors in PVN.^9,10^ Raphe dorsal nucleus afference to PVN regions and the significant density of 5-HT receptors in these hypothalamic nuclei have been described by several authors, and these results show that neuroendocrine responses to volume alterations may be modulated by 5-HT activating 5-HT_1A_ and/or 5-HT_2A_ receptors in the PVN.^11,12^

Based on the neuro-anatomical evidence of PVN oxytocinergic projections to NST and in works that have demonstrated serotonin neuromodulation over Oxt secretion, which is increased during isotonic BVE, we aim to evaluate serotonergic neuromodulation over 5-HT_2_ receptors in cardiovascular responses of the PVN in basal conditions or induced by BVE.

## Methods

### Animals

Thirteen male Wistar rats, weighing between 250-300 g in the beginning of the experiments were obtained and kept in the vivarium *Biotério da Disciplina de Fisiologia* at the University *Universidade Federal do Triângulo Mineiro* (UFTM), acclimatized to the controlled room temperature of 23 ± 2°C, with a light-dark cycle of 12h (light - 7 a.m. to 7 p.m.), with food and water *ad libitum*. These animals were divided into two groups: Control (N=8), and DOI (N=5). All experiments were done between 8 a.m. and 1 p.m., and were previously approved by the Ethics Committee on Animal Experimentation (CEUAUFTM) under protocol number 273.

### Habituation of the animals to experimental procedures

To decrease the influence of stress promoting factors at the time of experiments, the rats were handled daily and trained, for seven days, with the maneuvers used in the experimental protocol, such as: cleaning of the cannula and soft massage in the supra-pubic region for at least one week before the experiment.

### Experimental protocol

#### Cannulas in the PVN

The rats were anaesthetized with tribromoethanol (150mg/kg) and fixed to a stereotaxic device (*Insight Equipamentos - model ETX3/99, São Paulo, Brazil).* Two anthropometric points of the skull - bregma (union point of sagittal and coronal sutures) and lambda (union point of sagittal and lambdoid sutures) - were used as a reference to level the animals' heads in the horizontal plane. The the bregma, we determined the points for the bilateral introduction of the cannula in the rats's PVN. In these points, we made trepanations of the skull bones with a spherical drill, by opening two orifices of approximately 1.5 mm in diameter. In the PVN, stainless steel cannulas (12 x 0.55 mm d.i) were bilaterally positioned in the brain per the coordinates: 1.2 mm caudal to bregma; 0.5 mm lateral to the median line; and 5.0 mm under the dura-meter, according to the coordinates from the Atlas by Paxinos and Watson.^13^ The cannulas were positioned 2 mm above the PVN, and fixed to the skull with screws and acrylic dental resin. Metal chucks (0.3 mm d.i) were used to obliterate the cannulas. The rats received prophylactic injections of penicillin (20,000 units, i.m.). During the six days of recovery, before cannulation of the veins and femoral arteries, the rats were handled and trained daily for the procedure and cleaning of the chucks to reduce possible influences of stress responses due to animal manipulation.

### Cannulation of femoral veins and arteries

For the cardiovascular record of the conscious animals, on the day before the experiment, the animals were anaesthetized with tribromoethanol (150 mg/kg) for the implantation of polyethylene catheters (PE-50 and PE-10) in the abdominal aorta through the femoral artery to record the BP, and in the femoral vein to perform the BVE. After implantation, the cannulas were properly filled with a physiological solution and subcutaneously exteriorized in the posterior region of the neck. Before starting to record, the cannulas were heparinized (heparin 2% in physiological solution) to avoid the formation of clots.

### Cardiovascular records

After 24 hours of surgical recovery, the cannulas were washed with heparinized saline solution (0.1 mL of heparin sodium 25000 UI, Liquemine®, Roche, Rio de Janeiro, Brazil, dissolved in 20 mL of saline 0.9%). The arterial catheter was connected to a PA transducer (P23Db, Gould-Statham), and the signs of pulsatile BP were recorded in basal conditions for 30 minutes, and the signal was converted by an analogue-digital board (CODAS, with a sample frequency - 4 kHz, Di220 Dataq Instruments, Inc., Akron, OH, USA). During the experimental procedure, MBP and HR were derived from the pulsatile BP. During the recording, the animals stayed in a room with noise control, at a temperature of 27°C. After positioning of the animals and connection to the equipment, there was a 15-minute adaptation period before recording began. After adaptation of the animals and adequation of signal caption, we began the continuous recording of pulsatile BP for 30 minutes to obtain basal values of BP and HR.

### Microinjections of saline or drug in the brain

Thirty minutes after recording began, saline (1.0 *µ*g/200 ηL; n=8 animals) or serotoninergic dimethoxy-4-iodoamphetamine hydrochloride (DOI - 1.0 *µ*g/200 ηL; n=5 animals) dissolved in physiological saline solution were bilaterally injected in the PVN of the rats using a Hamilton syringe (5 *µ*L) connected by a PE-10 polyethylene tube to an injection needle introduced into the brain by the guide cannula, previously fixed to the brain. During and after the intracerebroventricular microinjections, animal records were done in a period of 30 minutes.

### Blood volume expansion (BVE)

Sixty minutes after beginning the recording, some animals underwent BVE, performed through intravenous infusion (femoral vein) of isotonic NaCL (0.15 M) in a volume of 2 ml/100 g of body weight during 60 seconds. During and after BVE, animal records were done in a 15-minute period.

### Recording of pulsatile BP

**Figure 1:**
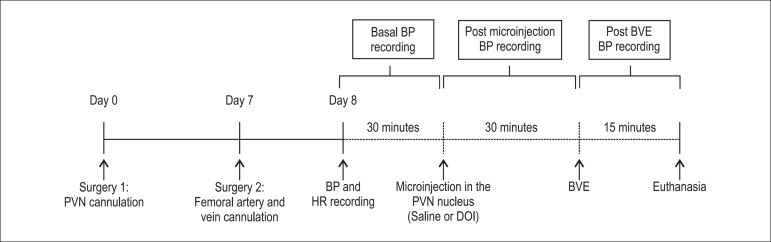
Representative scheme of the seven days of experimental protocol of the groups Control and Serotoninergic Agonist (DOI). PVN: paraventricular nuclei of the hypothalamus; BP, pulsatile BP; HR, BVE HR, extracellular volume expansion with isotonic saline (supplementary figure).

On the day of the experiment, between 8 and 9 a.m., the animals were weighed and the arterial cannula was connected to a pressure transducer; basal pulsatile BP was recorded for 30 minutes. After this period, the animals received microinjections of DOI in the PVN (1.0 *µ*g/200 ηL; n=5 animals) or the same volume of vehicle (isotonic saline; n=8 animals). After 30 minutes, the animals underwent isotonic BVE (NaCl 0.15 M/ 2 ml100 g weight), and the pressure was continuously recorded for another 15 minutes (Figure 1) (supplementary figure). After 75 minutes of recording, the animals were euthanized with thiopental sodium (100 mg/Kg) and their brains were removed and fixed in 10% formalin for a few days. Cross sections (40 *µ*m thick) were done in the points of PVN injection with a freezing microtome (MICROM, model HM 5000 M). Histologic sections, assembled onto slides, were stained by the Nissl method and analysed for PVN injection points according to the Atlas by Paxinos and Watson.^13^

### Study of the variability of BP and HR

Pulsatile BP was processed by a specific software that determined, beat-by-beat, SBP and HR values. HR, SBP, and DBP variability was also evaluated in the frequency domain, with the autoregressive spectral analysis method.^14,15^

Pulse Intervals (IP), SBP, and DBP time series, collected during 30 basal minutes, were divided into serial segments of 300 beats, and all successive segments overlapped in 50% with the previous segment (Welch method). Using stationary segments of the time series, autoregressive parameters were estimated through the Levinson-Durbin method, and the model order was chosen according to Akaike's criterion.^15^ After that, over each individual stationary segment of 300 beats, spectral decomposition was done. Normalization of values minimizes interference of the total potency over the components; normalization procedure was done by diving the potency of the low frequency component (LF - 0,15-0,4 Hz) or the high frequency component (HF - 0,04-0,15 Hz) by the total spectral potency, from which we subtracted the potency of the very low frequency band (VLF - 0,01-0,20 Hz), and then multiplied the result by 100.^15^ Spectral parameters obtained for each individual stationary segment of 300 beats were measured, and mean result values for the 30 basal minutes were collected for each animal. Quotient between LF and HF (LF/HF ratio) was used to express the sympathetic-vagal balance.^16^

### Statistical analysis

Statistical analysis was done through the software R, version 3.3.0. The obtained results were presented as mean ± standard deviation of the mean. To confirm that all continuous variables were normally distributed, we used the Kolmogorov-Smirnov test, and afterwards, to evaluate the effects of groups and evaluations in relation to SBP, DBP, MBP, HR, and LF/HF variables we used the two-way ANOVA with measurements, and Bonferroni's method of multiple comparisons. Significance level was set at 5%.

## Results

**Figure 2:**
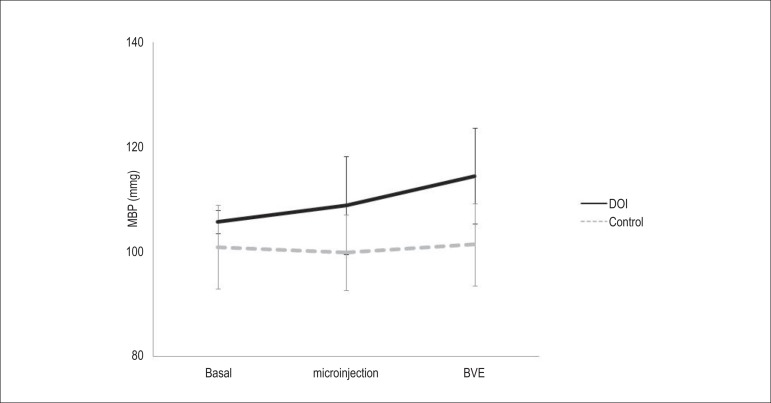
Mean values (± standard deviation of the mean) of the MBP of the basal control group animals (Cb), control after microinjection of saline (Cm), control after extracellular volume expansion (Ce) and those treated with DOI, in the basal state (Db), after DOI microinjection (Dm), and after extracellular volume expansion (De). #p=0.018 vs MBP Ce; ɤp=0.016 vs MBP Db.

Table 1 shows the results (mean ± standard deviation of the mean) of the cardiovascular variables of the control group animals (C) and of the DOI group animals (D). No significant differences were observed in the control group between the values obtained in basal period (Cb), after microinjection with saline (Cm), and saline followed by isotonic BVE (Ce) in the variables MBP, SBP, and DBP. DOI microinjection (Dm) and DOI followed by BVE (De) significantly increased MBP (Figure 2), SBP, and DBP in relation to the control group (Ce) (114.42±7.85 vs 101.34±9.17; 147.23±14.31 vs 129.39±10.70; and 98.01±4.91 vs 87.31±8.61, respectively).

**Table 1:** Mean values (± standard deviation of the mean) of mean blood pressure (MBP), systolic blood pressure (SBP), and diastolic blood pressure (DBP), heart rate (HR), low frequency component (LF), and high frequency component (HF) of the animals of the basal control groups (Cb), post saline microinjection in the paraventricular nuclei of the hypothalamus (Cm), control after expansion of the extracellular volume (Ce), and those treated with DOI, in the basal state (Db), after DOI microinjection in the paraventricular nuclei of the hypothalamus (Dm), and after expansion of the extracellular volume (De)

Variables	Cb	Cm	Ce	Db	Dm	De
MBP (mmHg)	100.83 ± 7.98	99.79 ± 7.24	101.34 ± 9.17	105.65 ± 2.25	108.79 ± 9.31	114.42 ± 7.85^#^^ɤ^
SBP (mmHg)	130.28 ± 7.62	129.66 ± 6.49	129.39 ± 10.70	136.05 ± 2.74	141.11 ± 14.95	147.23 ± 14.31^&^*
DBP (mmHg)	86.10 ± 8.53	84.86 ± 7.97	87.31 ± 8.61	90.46 ± 3.63	92.63 ± 6.50	98.01 ± 4.91^+^^%^
HR (bpm))	354.14 ± 29.53	356.14 ± 32.09	356.35 ± 41.99	399.40 ± 25.09^$^	405.08 ± 41.09^α^	421.02 ± 43.32^β^
LF/HF ratio	0.36 ± 0.20	0.55 ± 0.22	0.27 ± 0.32	0.67 ± 0.68	2.45 ± 0.82**	2.32 ± 0.80^##^

# p=0.018 vs MBP Ce;

ɤ p=0.016 vs MBP Db;

& p=0.010 vs SBP Ce;

* p=0.035 vs SBP Db;

+ p=0.047 vs DBP Ce;

% p=0.022 vs DBP Db;

$ p=0.034 vs HR Cb;

α p=0.033 vs HR Cm;

β p=0.010 vs HR Ce;

** p=0.001 vs LF/HF ratio Cm and Db;

## p=0.001 vs LF/HF Ce eDb.

Animals in the control group presented significant difference in basal HR values (Cb), with microinjection of saline (Cm) and saline followed by BVE, in relation the DOI group with the same treatments (Db, Dm, and De) (354.14±29.53 vs 399.40±25.09; 356.14±32.09 vs 405.08±41.09 and 356.35±41.99 vs 421.02±43.32, respectively). DOI microinjection (Dm), and DOI followed by BVE (De), increased the LF/HF ratio in relation to control animals who received saline microinjection (Cm) and saline followed by isotonic BVE (Ce) (2.45±0.82 vs 0.55±0.22 and 2.32±0.80 vs 0.27±0.32, respectively).

All p values obtained from statistical analysis of the studied variables are depicted in Table 2 (supplementary data).

**Table 2:** p values obtained after comparisons between the groups Control (n=8) and DOI (dimethoxy-4-iodoamphetamine) (n=5) of the studies variables: mean blood pressure (MBP), systolic blood pressure (SBP), and diastolic blood pressure (DBP), heart rate (HR), low frequency component (LF), and high frequency component (HF) of the animals of the basal control groups (Cb), post saline microinjection in the paraventricular nuclei of the hypothalamus (Cm), control after expansion of the extracellular volume (Ce), and those treated with DOI, in the basal state (Db), after DOI microinjection in the paraventricular nuclei of the hypothalamus (Dm), and after expansion of the extracellular volume (De). Variance analysis with repeated measurements and Bonferroni’s multiple comparison method were employed in this study. Significance level was set at p<0.05

Comparisons	MBP	SBP	DBP	HR	LF/HF
Cb vs Cm	0.827	0.937	0.568	0.987	0.704
Cb vsCe	0.828	0.930	0.568	0.988	0.755
Cm vs Ce	0.820	0.934	0.560	0.980	0.704
Db vs Dm	0.293	0.234	0.418	0.730	0.001
Db vs De	0.016	0.035	0.022	0.503	0.001
Dm vs De	0.096	0.230	0.075	0.500	0.711
Cb vs Db	0.287	0.316	0.308	0.034	0.290
Cm vs Dm	0.077	0.076	0.110	0.033	0.001
Ce vs De	0.018	0.010	0.047	0.010	0.001

## Discussion

Our study showed that isotonic BVE did not promote alterations in MBP, SBP, and DBP, or in HR and sympathetic-vagal ratio (LF/HF). As previously shown by other authors, acute BVE induces a series of hemodynamic events, including an increase in central venous pressure, right atrial pressure, central and peripheral blood volume, cardiac debit, and systolic volume. On the other hand, HR decreases significantly, and total peripheral resistance decreases slightly during volume overload, while MBP remains unaltered.^17^ These findings differ from those obtained by Godino et al., 2005, who performed BVE through infusion of a great intra atrial volume for one minute and observed a reduction not only in HR, but also in MBP. This hypotension can be mediated by a quick and pronounced release of oxytocin and atrial natriuretic peptide, which have a diuretic and vasodilatory effect.^18^ Moreover, these peptides are involved in the baroreflex control of the HR, facilitating vagal response in the increase of reflex bradycardia during baroreceptor discharge.^19-23^

In the present work, the animals who received intracerebroventricular microinjection of the serotonergic agonist DOI in the PVN, followed by BVE, presented a significant increase in MBP, SBP, DBP, HR, and LF/HF, suggesting that the serotonergic agonist DOI leads to the inhibition of Oxt secretion as a response to BVE, when it is bilaterally microinjected into the PVN, or even that is exerts a neuromodulation in the PVN level, which then promotes an inhibition in the baroreflex response to BVE.

Intravenous administration of 8-OHDPAT (5-HT_1A_ receptor agonist) or DOI (5-HT_2A_ receptor agonist) promotes an increase in plasma concentrations of Oxt. Both responses are significantly attenuated when the animals receive intravenous pre-treatment with antagonists of these receptor, suggesting that this increase occurs by the serotoninergic activation of these receptors, instead of stimulation by interneurons.^11,12,24,25^ 5-HT_2A_ receptors stimulation in the central nervous system can induce an increase in BP, partly through the increase in vasoconstrictor sympathetic activity due to sympathetic premotor neuron activation in the rostral ventrolateral medulla oblongata, and also through vasopressin release.^26^

PVN is reciprocally connected to several other areas of the brain involved in the cardiovascular function control.^27^ PVN also contains pre-autonomic neurons, which directly and indirectly project to sympathetic preganglionic neurons inside the spinal cord mediolateral cell column, via the rostral ventrolateral medulla oblongata.^28^

Several studies have reported the contribution of PVN parvocellular neurons in the compensatory autonomic response during physical training for volume overload, suggesting that the volume overload stimulates vagal cardiac receptors, especially by the activation of PVN parvocellular neurons, which successively induces the inhibition of the sympathetic nervous activity.^29-35^ Several works have suggested that the increase in neuron activity in the PVN is associated to sympathetic excitation during cardiac collapse.^36-38^ Moreover, some works have found that an altered GABAergic mechanism in the PVN may be involved in the regulation of the sympathetic afferent in the cardiac collapse, and that alterations in the inhibitory mechanism can contribute to an increase in sympathetic activity.^39-40^

This is the first study to explore serotoninergic neuromodulation via 5-HT^2A^ receptors in the PVN level about cardiovascular responses to isotonic BVE, which proved to be inhibitory. However, it is noteworthy that further, more in depth studies are necessary to check if this neuromodulation directly affects sympathetic and/or baroreflex activity or if it is accompanied by neuroendocrine alterations, especially concerning Oxt, arginine, vasopressin, and atrial natriuretic peptide secretion.

## Conclusion

The present work provides evidence that serotonin performs neuromodulation in the PVN level, which promotes an inhibition of the baroreflex response to BVE. Thus, this work suggests the serotoninergic involvement in the neuromodulation in the PVN level in the vagal reflex response in normotensive rats.
